# A fully automated AI-based method for tumour detection and quantification on [^18^F]PSMA-1007 PET–CT images in prostate cancer

**DOI:** 10.1186/s40658-025-00786-9

**Published:** 2025-08-20

**Authors:** Elin Trägårdh, Johannes Ulén, Olof Enqvist, Måns Larsson, Kristian Valind, David Minarik, Lars Edenbrandt

**Affiliations:** 1 Department of Translational Medicine, Wallenberg Centre for Molecular Medicine, Lund University, Malmö, Sweden; 2 Department of Clinical Physiology and Nuclear Medicine, Skåne University Hospital, Inga Marie Nilssons G 47, 205 02 Malmö, Sweden; 3 Eigenvision AB, Malmö, Sweden; 4 Department of Electrical Engineering, Chalmers University of Technology, Gothenburg, Sweden; 5 Department of Radiation Physics, Skåne University Hospital, Malmö, Sweden; 6 Department of Clinical Physiology, Sahlgrenska University Hospital, Gothenburg, Sweden; 7 Department of Molecular and Clinical Medicine, Sahlgrenska Academy, University of Gothenburg, Gothenburg, Sweden

**Keywords:** Prostate cancer, PET–CT, Artificial intelligence, PSMA, CNN

## Abstract

**Background:**

In this study, we further developed an artificial intelligence (AI)-based method for the detection and quantification of tumours in the prostate, lymph nodes and bone in prostate-specific membrane antigen (PSMA)-targeting positron emission tomography with computed tomography (PET–CT) images.

**Methods:**

A total of 1064 [^18^F]PSMA-1007 PET–CT scans were used (approximately twice as many compared to our previous AI model), of which 120 were used as test set. Suspected lesions were manually annotated and used as ground truth. A convolutional neural network was developed and trained. The sensitivity and positive predictive value (PPV) were calculated using two sets of manual segmentations as reference. Results were also compared to our previously developed AI method. The correlation between manually and AI-based calculations of total lesion volume (TLV) and total lesion uptake (TLU) were calculated.

**Results:**

The sensitivities of the AI method were 85% for prostate tumour/recurrence, 91% for lymph node metastases and 61% for bone metastases (82%, 86% and 70% for manual readings and 66%, 88% and 71% for the old AI method). The PPVs of the AI method were 85%, 83% and 58%, respectively (63%, 86% and 39% for manual readings, and 69%, 70% and 39% for the old AI method). The correlations between manual and AI-based calculations of TLV and TLU ranged from r = 0.62 to r = 0.96.

**Conclusion:**

The performance of the newly developed and fully automated AI-based method for detecting and quantifying prostate tumour and suspected lymph node and bone metastases increased significantly, especially the PPV. The AI method is freely available to other researchers (www.recomia.org).

**Supplementary Information:**

The online version contains supplementary material available at 10.1186/s40658-025-00786-9.

## Background

Prostate cancer is one of the most common cancers and one of the most common causes of cancer deaths among men worldwide [1]. For correct management, risk stratification is crucial. Imaging plays an important role in the work-up of prostate cancer. Multiparametric magnetic resonance imaging is often performed in patients with elevated prostate-specific antigen (PSA) levels and has been found to accurately select who will benefit from a prostate biopsy [2]. In patients with a confirmed high-risk disease or with a rising PSA after a curative treatment, other imaging methods are available. The introduction of prostate-specific membrane antigen (PSMA)-targeted positron emission tomography (PET) imaging has improved the detection of loco-regional and metastatic disease [3–7]. In recent years, it has emerged as the preferred method for staging newly diagnosed high-risk prostate cancer, finding sites of recurrence and evaluating eligibility for treatment with [^177^Lu]Lu-PSMA-617 and analogues [8–10].

For diagnostic purposes, several different PSMA agents have been introduced, such as [^68^Ga]Ga-PSMA-11, [^18^F]DCFPyL and [^18^F]PSMA-1007 [11–14]. The widespread adoption of PSMA PET–CT has significantly increased the workload for nuclear medicine departments and nuclear medicine physicians. Artificial intelligence (AI) algorithms, particularly deep learning such as convolutional neural networks (CNN), have demonstrated progress in image-recognition tasks. Integrating an AI application within the imaging workflow could potentially increase efficiency, reduce errors and reduce inter-individual variations [15–17].

Previously, we developed a fully automated AI-based method that can detect and quantify prostate cancer-related tumours and metastases in PSMA PET–CT scans [18, 19]. Our previous studies showed that the AI-based method obtained a sensitivity on par with that of nuclear medicine physicians. However, the AI-based method had a higher number of false positive lesions compared to nuclear medicine physicians, especially for the detection of suspected lymph node metastases (on average 2.8 false positive lesions per patient for the AI-based method and 0.5 for physicians) and bone metastases (on average 3 false positive lesions per patient for the AI-based method and 1.8 for physicians). The correlations of total lesion volume (TLV) and total lesion uptake (TLU) between AI-based and manual calculations were moderate to strong. Previous studies were performed with a training and validation group of 540 and 120, respectively. We hypothesise that the performance of the AI-based method can be improved with a larger training group and alterations in the CNN method.

The aim of the present study was to develop an AI-based method for detecting and quantifying tumour lesions (prostate tumour, prostate recurrence, as well as metastases in lymph nodes and bone) in [^18^F]PSMA-1007 PET–CT images and to compare the results to experienced nuclear medicine physicians. Secondary aims were to address the individual contributions of the additional data and the newly developed training pipeline, and to make the AI method freely available for future research.

## Methods

### Patients

Patients admitted for clinically indicated [^18^F]PSMA-1007 PET–CT to Department of Clinical Physiology and Nuclear Medicine, Skåne University Hospital, Lund or Malmö, Sweden, were eligible for inclusion. Indications for PSMA PET–CT referrals at our hospital included primary staging of high-risk prostate cancer or secondary staging after biochemical recurrence. The lower PSA limit for PSMA PET–CT referrals due to biochemical recurrence at our hospital was 0.2 ng/ml. A total of 1064 patients, who were scanned from December 2019 to December 2021, were included. All 660 patients from the study conducted by Tragardh et al. [19] were included as well as an additional 404 consecutive patients between May 2021 and December 2021. The study was approved by the ethics committee at Lund University (#2016/417, #2018/753 and #2021–05734-02) and followed the principles of the Declaration of Helsinki. All patients provided written informed consent.

### Imaging

Patients were injected with 4 MBq/kg of [^18^F]PSMA-1007 and after two hours imaged on a Discovery MI PET–CT (GE Healthcare, Milwaukee, WI, USA) from the base of the skull to the mid-thigh. The acquisition time was two minutes per bed position. Images were reconstructed with a block-sequential regularisation expectation maximisation algorithm (Q.Clear; GE Healthcare, Milwaukee, WI, USA) with a beta factor of 800 [20]. Time-of-flight, point spread function modelling, a 256 × 256 matrix with pixel size of 2.7 × 2.7 mm^2^ and a slice thickness of 2.8 mm were used. Either a low-dose or a diagnostic CT with oral and intravenous contrast was performed and used for attenuation correction and anatomic correlation.

### Manual segmentations for training

Two experienced nuclear medicine physicians segmented a suspected prostate tumour or local recurrence, suspected lymph node metastases and suspected bone metastases in the PSMA PET–CT images. One of the readers segmented 921 of the scans, and the other reader segmented the remaining 143 scans. The cloud-based annotation platform RECOMIA (www.recomia.org) was used for the manual segmentations. The RECOMIA platform includes basic display features for PET–CT images and segmentation tools [21]. Of the full set of 1064 studies, 120 were used as a test set and 120 as a validation set. The test and validation sets were the same as in the Tragardh et al. study [19]. In the test set, approximately 60% of the patients were referred due to primary staging and 40% due to biochemical recurrence. The remaining 824 studies were used as the training set.

### AI model

At the core of our AI solution, a four-level U-Net [22] was implemented and trained using the MONAI framework [23]. Inference and training were both performed on patches of size 192 × 192 × 192 pixels.

Input to the network consisted of the CT image and the SUV image calculated from the PET image. Both images were resampled to a pixel size of 1.33 × 1.33. × 3.0 mm, and intensities were clamped and normalised to [0, 1]. The CT image was clamped to [–1024, 3072], and the SUV image was clamped to [0, 100] before normalisation.

Compared to the Tragardh et al. study [19], the most important differences were.

- *Deeper U-Net* Using a deeper U-Net allows for a larger receptive field and more powerful modelling capacity.
- *Same padding* Zero-padding the input of each convolutional layer such that the output retains the same spatial dimensions as the input. This typically leads to more efficient training and much faster inference.
- *Deep supervision* An auxiliary loss was applied to lower levels of the network.

### Sampling

For each training image, a sample mask, where each foreground class was weighted equally, was constructed. In addition, the total weight of all foreground pixels was equal to the total weight of all background pixels. In other words, each foreground sampled equally often, and half of the samples were background samples.

All pixels within each class were sampled at the same frequency. However, after each 20-epoch interval, the sampling was updated based on the pixelwise loss of the current model. This was done assure that ‘hard’ background pixels were sufficiently sampled. More specifically, 50% of the training images were randomly selected and had their sample masks updated. For the loss sample mask, all background pixels were weighted proportionally to the pixelwise loss up to an allowed maximum value (500 times the average sample probability). The new sample mask was then created by taking the average of the old sample mask and the loss sample mask in each pixel. This enabled more sampling of the background pixels where the model struggled while keeping the foreground sampling and the foreground to background sampling ratio fixed.

Note that the samples drawn from the sample mask specified the centre pixel of the input patch. This means that even though a foreground pixel was sampled, the corresponding patch contained many background samples.

### Training

The model was trained using mixed half precision on an NVIDIA RTX 3090 GPU. During training, an epoch was defined as 10,000 samples and the model was trained for 200 epochs. The model was optimised using Nadam [24] with an initial learning rate set to 5 × 10^–5^. The learning rate was multiplied by 0.985 after each completed epoch. Weighted categorical-cross entropy was used as a loss function with weight 1.0 for background and 25.0 for foreground. In addition, deep supervision [25] was applied to lower levels of the network with weights of 0.5, 0.25 and 0.125.

Every 40 epochs, a model was saved, and the model with the best performance on the validation set was chosen (the one that had trained for 80 epochs).

### Augmentation

As with most medical applications, data was limited. To address this limitation and enhance the dataset's variability as well as the resulting model’s robustness, data augmentation was used. The patches were spatially augmented by applying a randomised affine transformation with the following randomisation ranges.

- *Scaling*: − 20% to 20%.
- *Rotation*: up to 15°.
- *Shear*: − 0.05 to + 0.05.
- *Translation*: move center to anywhere in input patch.

Additionally, the CT intensities were randomly augmented:

- 25% chance to apply smoothing using a Gaussian kernel with standard deviation drawn uniformly from the range 0.25 to 1.0 for each dimension.
- 50% chance to add Gaussian noise. The mean value for the distribution was drawn uniformly from − 50 to 50 and the standard deviation was drawn uniformly from 0 to 100.
- 25% chance to apply intensity scaling. The intensities were scaled with a factor drawn uniformly from [0.95, 1.05]

Note that each probability was independent, and that some patches received no intensity augmentation.

### Inference

Inference was performed using a sliding window with the same input patch size as for the training, i.e. 192 × 192 × 192 pixels. With same padding, an output was produced for each pixel in the input patch, but results for pixels close to the patch boundary were less reliable, as their receptive field was reduced. Hence input patches generated with an overlap of 94 pixels, and for pixels where multiple outputs were computed, the one with the largest receptive field was used.

The average inference time on the test set images were 42.5 s on a computer with a NVIDIA RTX 3090 GPU and an AMD Ryzen 9 5900X 12-Core Processor.

### Reference model training

For comparison, a nnU-Net model [26] was also trained on the same dataset using the publicly available training pipeline. Initial training with the default "3d_fullres" configuration led to rapid convergence to a degenerate solution that predicted only background. To address this, a modified training strategy was employed: the model was first pretrained on a small, handpicked subset of the data consisting exclusively of studies containing all target classes and a lot of foreground. This pretrained model was then used to initialize the full training. Additionally, the “oversample foreground” percent parameter was increased to 0.66 to further mitigate the risk of background-only predictions. Dice score, sensitivity and PPV were compared.

### Ablation study of pipeline and data contributions

An ablation study was conducted to assess the individual contributions of the newly developed training pipeline and the expanded training dataset. Three configurations were evaluated:

1. Baseline model, as described in Trägårdh et al. [19], using the training pipeline and dataset from their paper,
2. Pipeline-only model, in which the new training pipeline was applied to the dataset from [19],
3. Model trained on the expanded dataset with the new training pipeline.

Through this setup, the relative contributions of the training pipeline improvements and data expansion were isolated and quantified. Dice score, sensitivity and PPV were compared.

### Model evaluation

To compare the results of this updated AI-based method to previous results, we employed a similar evaluation method as described by Tragardh et al. [19]. The performance of the AI-based method was assessed using the test set of 120 patients and two sets of ‘expert readers’.

Reading A was conducted by an experienced nuclear medicine physician who also performed most of the manual segmentations for the model training. Reading B was conducted by five other physicians (three board certified nuclear medicine physicians and two residents in nuclear medicine), each segmenting suspected tumours and metastases in 24 cases from the test set. Their experience with PET–CT readings ranged from 5 to over 10 years. The readers were instructed to mark all suspected malignant lesions in the prostate and seminal vesicles in patients with a prostate, and suspicious recurrence in the prostate bed in patients without a prostate (after prostatectomy). They also marked suspected lymph node metastases and bone metastases, using the E-PSMA grading system as guidance [27].

The AI-based method was evaluated on a lesion-based level (see below), focusing on suspected prostate tumour/recurrence, suspected lymph node metastases and suspected bone metastases. To assess inter-reader variability, comparisons were made between the expert readers. Readings A and B were alternately used as the reference (‘gold standard’) and compared to either the other human reading or the AI-based method.

True positive lesions were defined as lesions with either partial or full segmentation overlap with the reference reading. Lesions detected by the AI-based method or a human reading without segmentation overlap with the reference reading were regarded as a false positive. False negative lesions were defined as a lesion detected by the reference reading, but not by the AI-based method or a human reading. Sensitivity was calculated as the proportion of suspected lesions detected by a human reading or the AI-based method of those detected by the reference human reading. The positive predictive value (PPV) was calculated as the proportion of true positive lesions for a human reading or the AI method compared to the reference reading, divided by the sum of false positive and true positive lesions compared to the same reference reading.

Tumour burden was measured as TLV (cm^3^) and TLU (cm^3^). TLV was calculated by summing the volume of all positive voxels identified by manual or AI-based segmentations. TLU was calculated by multiplying the SUVmean by the TLV for each lesion and then summing all lesion TLUs to provide a total TLU.

### Statistical analysis

Sensitivity and PPV were calculated for the AI-based method and the human readings, as described above. The correlations of the tumour burden (TLV and TLU) measured by the AI model and Reading A were evaluated with Bland Altman plots, as well as with scatter plots and the Spearman rank correlation with a two-tailed test. A significance level of *p* = 0.05 was used. The correlation was considered very strong for Spearman’s coefficient r > 0.8, strong if the value was between 0.6 and 0.8, moderate for values between 0.4 and 0.6 and weak for values below 0.4.

For each lesion type the new AI model was also compared to the one from Trägårdh et al. [19] using two sign tests. For each image the number of false positives (negatives) for the two models provided a pair of observations. The sign test was used to test the hypothesis that the two models were equally likely to produce more false positives (negatives) for an image. A significance level of *p* = 0.05 was used. The statistical analysis was carried out in R, version 4.0.3.

## Results

### Detection of suspected tumour lesions

When all tumours/metastases were grouped together, Reading A noted a total of 622 suspected lesions (average 5.2 per patient), to be compared to 780 (6.5 per patient) for Reading B, 691 (5.8 per patient) for the AI method and 1293 (10.8 per patient) for the old AI method. The sensitivity for the AI method compared to Reading A was 85% and the PPV was 72%.

For prostate tumour/recurrence, Reading A detected suspected tumour(s) in 79 (66%) of the patients. On average, 0.8 prostate tumour/recurrence was detected per patient for Reading A. The sensitivity for the AI method for detecting prostate tumour/recurrence was 95% when Reading A was used as reference, while the PPV was 84%. This can be compared to a sensitivity of 94% and a PPV of 55% for our previously developed AI method. The new AI method had fewer false positives than the older method in 41 of the test images and more in three of the test images, which showed a significant difference (*p* < 0.001). The number of false negatives was equal in all test images.

For lymph node metastases, Reading A detected suspected metastases in 42 (35%) of the patients. On average, Reading A detected 2.1 lymph node metastases per patient. The sensitivity for the AI method was 90% compared to Reading A, and the PPV was 64%. This can be compared to a sensitivity of 88% and a PPV of 39% for our previously developed AI method. The new AI method had fewer false positives than the older method in 71 of the test images and more in 14 of the test images, which indicated a significant difference (*p* < 0.001). The difference in false negatives was not significant (*p* = 0.75).

For bone metastases, Reading A detected suspected metastases in 39 (33%) of the patients, corresponding to an average of 2.4 suspected bone metastases per patient. The sensitivity for the AI method was 74% and the PPV was 71% when Reading A was used as reference. In comparison, our previously developed AI method had a sensitivity of 88% and a PPV of 39%. The new AI method had fewer false positives than the older method in 63 of the test images and more in 13 of the test images, which was a significant difference (*p* < 0.001). The difference in false negatives was not significant (*p* = 0.55).

**Fig. 1:**
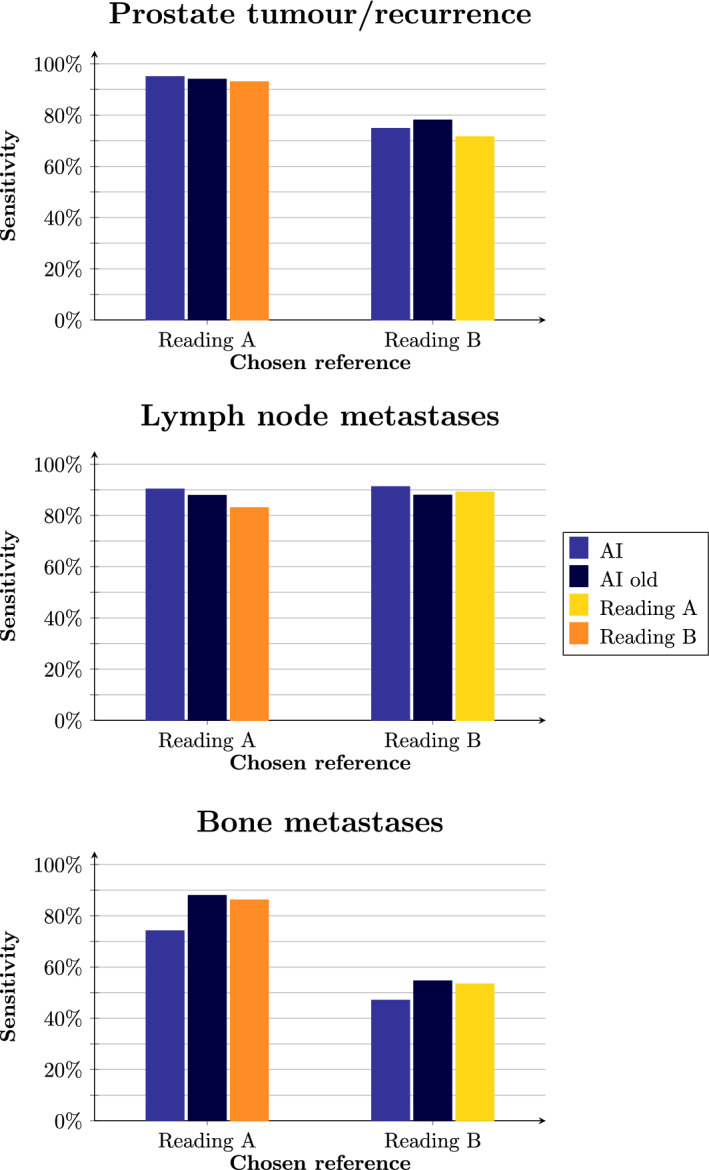
Sensitivity of the new and the old AI models as well as the human readings, when using Reading A and Reading B as references, respectively

**Fig. 2:**
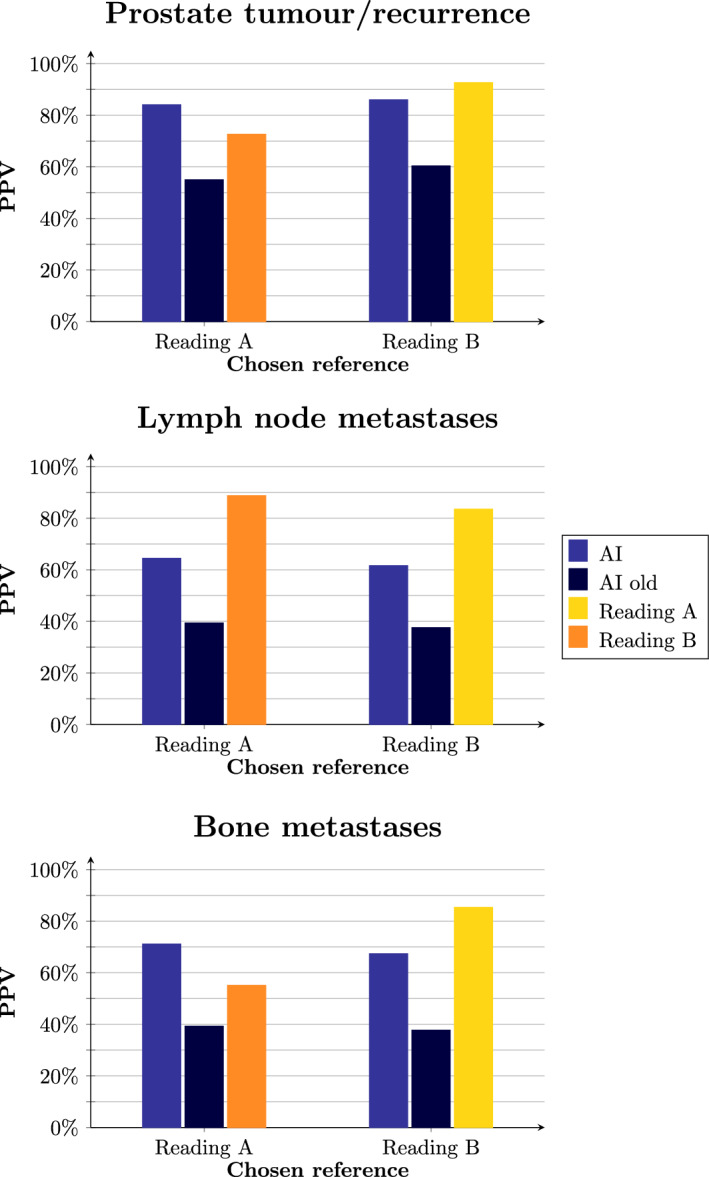
PPV of the new and the old AI models as well as the human readings, when using Reading A and Reading B as references, respectively

Comparisons of sensitivity and PPV for different locations for the new and old AI method as well as the human readings can be found in Figs. 1 and 2. The number of false positive lesions were considerably lower for the new AI method compared to the old, which is detailed in Tables 1, 2 and 3. The sensitivity for the new AI method was worse for bone metastases compared to the old AI method and the human readers. The new AI method had worse PPVs compared to human readings for detecting lymph node metastases and worse PPV compared to Reading A for prostate tumour/recurrence and bone metastases when Reading A was used as reference.

**Table 1:** Sensitivity, positive predictive value (PPV), true and false positive lesions (TP/FP), and false negative lesions (FN) for detection of prostate tumour/recurrence. Average is shown when one Reading at a time was used as reference

n = 120 patients	AI vs reading	Reading vs reading	Old AI vs reading
Sensitivity (%)	84.9%	82.3%	68.7%
PPV (%)	85.0%	82.6%	57.7%
TP (n)			
Total	93.5	90.5	95
Per patient	0.78	0.75	0.79
FP (n)			
Total	16.5	21	70
Per patient	0.14	0.18	0.58
FN (n)			
Total	18	21	16.5
Per patient	0.15	0.18	0.14

**Table 2:** Sensitivity, positive predictive value (PPV), true and false positive lesions (TP/FP), and false negative lesions (FN) for detection of suspected lymph node metastases. Average is shown when one Reading at a time was used as reference

n = 120 patients	AI vs reading	Reading vs reading	Old AI vs reading
Sensitivity (%)	90.7%	86.1%	87.9%
PPV (%)	63.0%	86.1%	38.5%
TP (n)			
Total	220.5	209	213.5
Per patient	1.84	1.74	1.78
FP (n)			
Total	129.5	34	341.5
Per patient	1.08	0.28	2.85
FN (n)			
Total	22.5	34	29.5
Per patient	0.19	0.28	0.25

**Table 3:** Sensitivity, positive predictive value (PPV), true and false positive lesions (TP/FP), and false negative lesions (FN) for detection of suspected bone metastases. Average is shown when one Reading at a time was used as reference

n = 120 patients	AI vs reading	Reading vs reading	Old AI vs reading
Sensitivity (%)	60.6%	69.8%	71.3%
PPV (%)	69.2%	70.2%	38.5%
TP (n)			
Total	204.5	235	240
Per patient	1.70	1.96	2.00
FP (n)			
Total	91	118.5	383.5
Per patient	0.76	0.99	3.20
FN (n)			
Total	149	118.5	113.5
Per patient	1.24	0.99	0.95

Table 4 presents a comparison between the AI method and the reference nnU-Net model. The nnU-Net model achieved higher PPV across all three classes. However, this came at the cost of low sensitivity, significantly lower compared to the proposed AI method.

**Table 4:** Dice score, sensitivity and PPV for detection of prostate tumour/recurrence, lymph node metastasis and bone metastasis. nnU-Net refers to results obtained using the standard nnUN-et configuration with default settings. nnU-Net* denotes a modified version, where adjustments were made to ensure convergence to a non-trivial solution

		AI vs reading	NnU-Net* vs reading	NnU-Net vs reading	Reading vs reading
Prostate	Dice	0.846	0.789	0	0.812
	Sensitivity (%)	84.9%	67.7%	0	82.3%
	PPV (%)	85.0%	95.4%	–	82.6%
Lymph node	Dice	0.744	0.760	0	0.860
	Sensitivity (%)	90.7%	65.2%	0	86.1%
	PPV (%)	63.0%	91.1%	–	86.1%
Bone	Dice	0.640	0.521	0	0.665
	Sensitivity (%)	60.6%	36.3%	0	69.8%
	PPV (%)	69.2%	95.3%	–	70.2%

Table 5 presents an ablation study conducted to evaluate the individual contributions of the additional training data and the newly developed training pipeline, in comparison to the approach presented by Trägårdh et al. [19]. Based on the component-wise Dice scores, the new training pipeline demonstrated improved performance over the original method. As expected, further performance gains were observed when the model was trained on the expanded dataset. It should be noted that the Dice scores reported were calculated at the component level and can be interpreted as the harmonic mean of positive predictive value (PPV) and sensitivity, thereby reflecting a balance between these two metrics.

**Table 5:** Dice score, sensitivity and PPV for detection of prostate tumour/recurrence, lymph node metastasis and bone metastasis. AI old data is a model trained on the training set of Trägårdh et al. [19] but with the training pipeline presented in this work. Note that these results are on the validation set

		AI vs reading	AI old data vs reading	Old AI vs reading
Prostate	Dice	0.866	0.798	0.727
	Sensitivity (%)	94.9%	91.9%	89.9%
	PPV (%)	79.7%	70.5%	61.0%
Lymph node	Dice	0.703	0.685	0.437
	Sensitivity (%)	87.2%	87.9%	90.1%
	PPV (%)	58.6%	56.1%	28.9%
Bone	Dice	0.705	0.637	0.511
	Sensitivity (%)	92.5%	95.8%	97.5%
	PPV (%)	56.9%	47.7%	34.6%

### Quantification of tumour burden

**Fig. 3:**
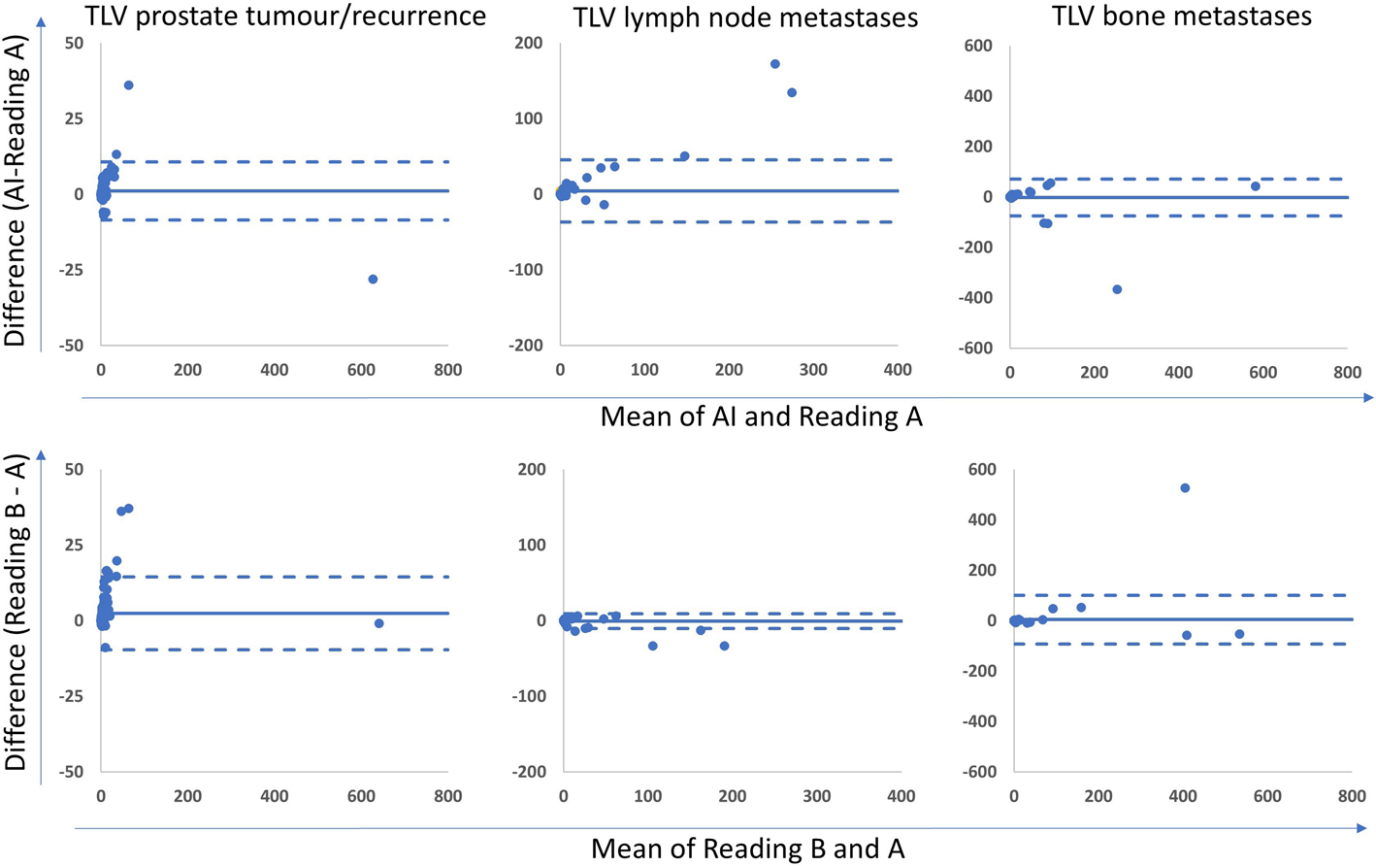
Bland Altman plots of the relationship between TLV in prostate tumour/recurrence, suspected lymph node metastases and bone metastases measured by the AI and human readings

**Fig. 4:**
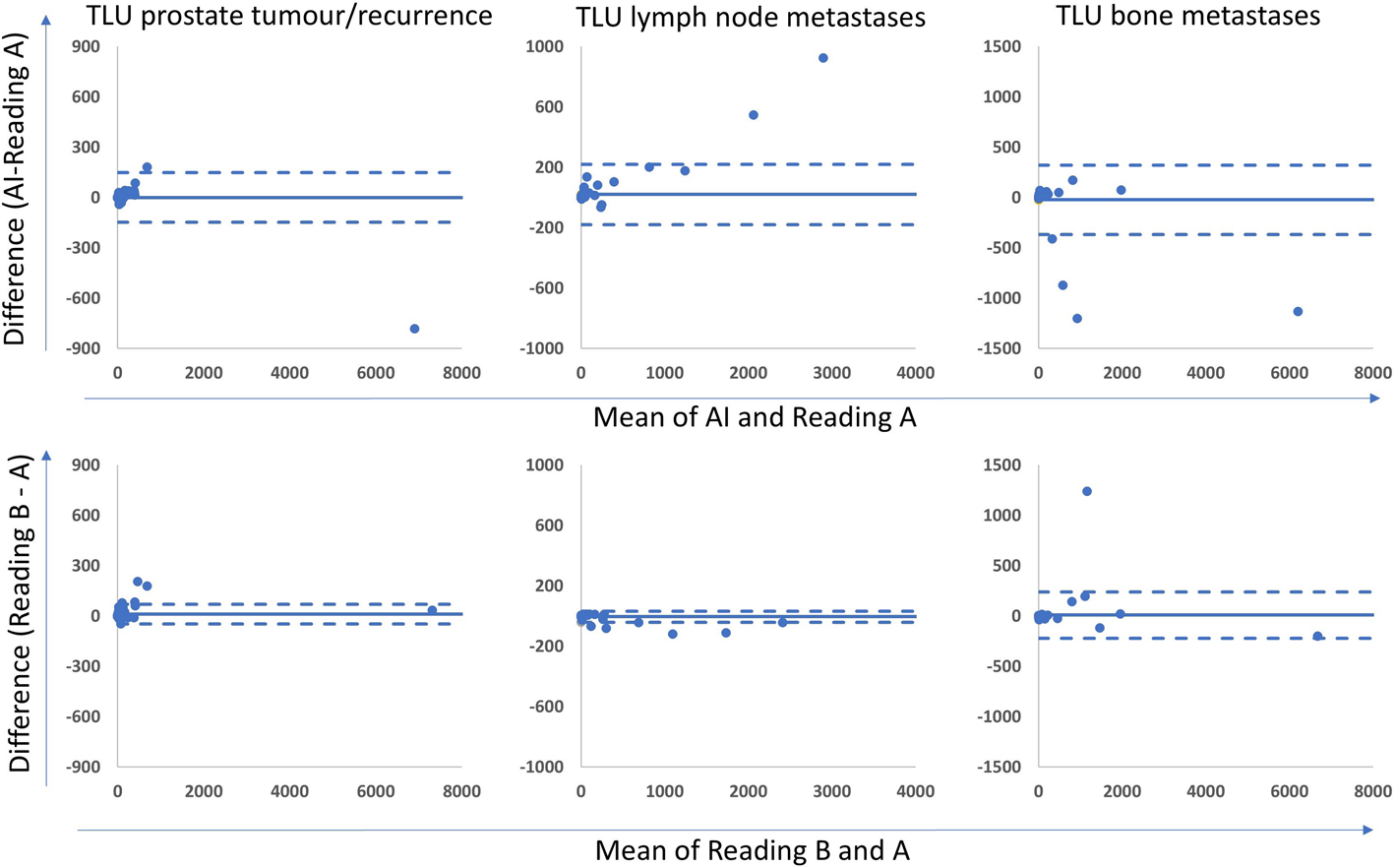
Bland Altman plots of the relationship between TLU in prostate tumour/recurrence, suspected lymph node metastases and bone metastases measured by the AI and human readings

**Fig. 5:**
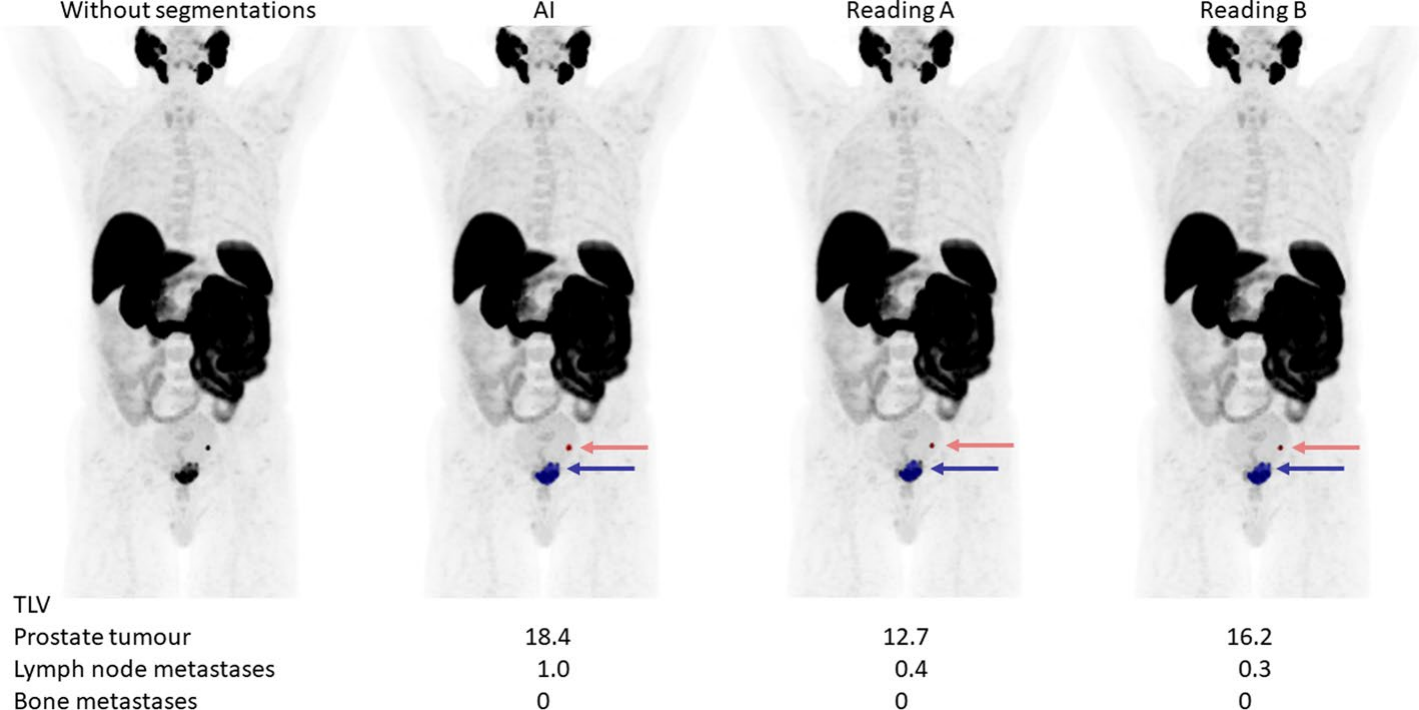
A patient example of segmentations performed by the AI method, Reading A and Reading B. Suspected tumours/metastases are indicated by the arrows. The TLV for the different tumour locations are noted

**Fig. 6:**
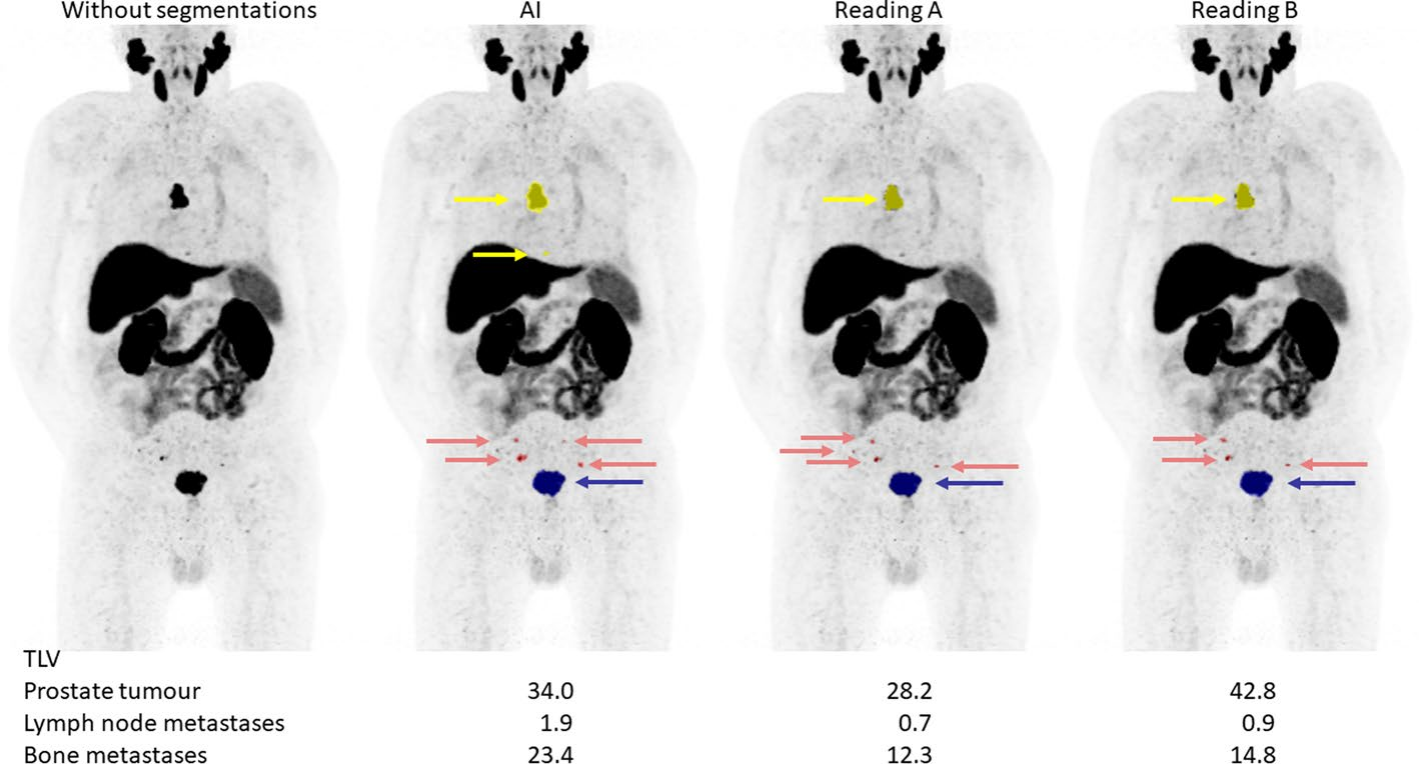
A patient example of segmentations performed by the AI method, Reading A and Reading B. Suspected tumours/metastases are indicated by the arrows. The TLV for the different tumour locations are noted

The tumour burden, measured as TLV and TLU, is shown in Table 6. The correlations for TLV and TLU between the new AI method and Reading A ranged between 0.73 and 0.96; for the AI method and Reading B between 0.62 and 0.93; and between the two manual readings between 0.76 and 0.93. All correlations were highly significant (*p* < 0.001) (Table 7). Scatter plots are shown in supplementary Figs. 1 and 2. Bland Altman plots are displayed in Figs. 3 and 4. The bias was close to 0 in all comparisons. Limits of agreement for TLV were smaller when comparing the AI method to reading A than when the manual readings were compared for both prostate lesions and bone lesions. Limits of agreement for TLU were wider when the AI method was compared with Reading A than when the manual readings were compared. Examples of segmentations performed by the AI method, Reading A and Reading B are shown in Figs. 5 and 6.

**Table 6:** Median (range) values of tumour burden, measured as TLV and TLU for the prostate tumour/recurrence, suspected lymph node metastases and bone metastases for the new and old AI, as well as for the two different manual Readings

	AI	Reading A	Reading B	Old AI
TLV Prostate tumour	1.7 (0–613)	1.2 (0–641)	2.0 (0–640)	1.6 (0–491)
TLV Lymph nodes metastases	0.2 (0–342)	0 (0–207)	0 (0–174)	0.8 (0–224)
TLV Bone metastases	0.2 (0–602)	0 (0–559)	0 (0–669)	0.5 (0–495)
TLU Prostate tumour	8.5 (0–6509)	6.4 (0–7291)	12.6 (0–7326)	11.3 (0–5903)
TLU Lymph nodes metastases	0.5 (0–3358)	0 (0–2433)	0 (0–2390)	4.0 (0–2810)
TLU Bone metastases	1.0 (0–5635)	0 (0–6768)	0 (0–6570)	1.5 (0–5199)

**Table 7:** Spearman correlation coefficients (r_s_) of the AI-method and the two manual readings

	AI vs reading A	AI vs reading B	Reading A vs B
TLV Prostate tumour	0.94	0.89	0.88
TLV Lymph node metastases	0.77	0.75	0.80
TLV Bone metastases	0.75	0.62	0.75
TLU Prostate tumour	0.96	0.93	0.93
TLU Lymph node metastases	0.76	0.75	0.83
TLU Bone metastases	0.73	0.61	0.76

### Discussion

In this study, we further advanced a fully automatic AI-based method for detection and quantification of tumour and suspected metastases on [^18^F]PSMA-1007 PET–CT images in patients with prostate cancer. The main results demonstrate that the sensitivity of the new model remained comparable to that of nuclear medicine physicians, while the PPV had significantly improved compared to our previously developed AI method [19]. For example, the false positive rate per patient for suspected lymph node metastases decreased from 2.85 to 1.08. Although the overall performance was better for the new AI method compared to the old, the sensitivities of the new AI method were worse for bone metastases compared to the old AI method and the human readers.

Recent systematic reviews have summarized the research on AI applications in PSMA PET–CT imaging for prostate cancer [28, 29]. Different attempts have been made with different purposes, ranging from detection of tumours and metastases, lesion classification, tumour quantification and prediction/prognostication, by using CNN or classical machine learning techniques, for example, on radiomics models. Approximately ten studies published in recent years have focused on AI-based methods for detecting and/or quantifying PSMA PET–CT images [29]. Overall, AI methods can detect suspected lymph node metastases and bone metastases with sensitivities ranging between 62 and 97%. The reported PPV in previous research is generally low and widely variable. The improved PPV of our new AI method compared to our previous study can be attributed to the larger training set, deep supervision and a larger field of view for the model. The ablation study (Table 5) indicates that the performance improvements over our previous method [19] result from both architectural and training pipeline enhancements, as well as the inclusion of additional training data.

Jafari et al. [30] recently developed an AI-based method that obtained a sensitivity of 88–95% and a PPV of 98–100% at a lesion level. Their study did not differentiate between the prostate tumour and metastases, but all tumour-related findings were marked using a single label. They found strong correlations between automated and manual measurements of TLV and TLU. Their study used [^68^Ga]Ga-PSMA-11 images and had mostly patients with recurrence (89% in the training group and 81–89% in the test groups, compared with approximately 40% in our test group). The number of negative scans in their study were very low (none in the training group, 11% in the test groups). In our test set, 12% were negative examples, but when regarding only patients without lymph node or bone metastases, we had 46% negative examples for Reading A and 49% for Reading B. Thus, the patient cohort and distribution of disease burden are not comparable between the studies. For their study, Jafari et al. [30] used the publicly available nnU-Net [26], an AI training pipeline, which is commonly used as an out-of-the-box solution to provide a baseline for comparison. The nnU-Net [26] training pipeline was also applied to the dataset used in the present study; however, initial training attempts were unsuccessful. This is probably due to the relatively high number of negative examples in the training set of this study. When trained on the full dataset using the default configuration, the nnU-Net rapidly converged to a solution in which all voxels were classified as background. With modifications to the training pipeline, convergence to a meaningful segmentation outcome was eventually achieved. Nevertheless, the sensitivity of the resulting model was unacceptably low. Attempts to improve sensitivity by adjusting the foreground probability threshold were ineffective, as the model's output probabilities were highly polarized, tending toward values close to zero or one for nearly all voxels.

To date, only one CE- and FDA-approved product exists for PSMA PET–CT interpretation, trained on [^18^F]DCFPyL PET and low-dose CT scans [31]. The software aPROMISE offers quantitative analysis of hotspots and standardised reporting of PSMA PET–CT scans. In a retrospective study [31], the sensitivity for detecting suspicious lesions was high, ranging between 87% (bone) and 92% (regional lymph nodes), using manually selected lesions as reference. The number of false positive lesions in this study was high, ranging from eight instances per patient for suspected bone metastases to 90 for suspected lymph node metastases. It is not known how the software performed on other PSMA radiopharmaceuticals or if a diagnostic CT with intravenous contrast was used.

Our AI tool is freely available for research at www.recomia.org. Another research group has also released an AI model for automatic segmentations of intraprostatic tumour lesions on [^68^Ga]Ga-PSMA PET–CT [32].

Quantitative metrics such as TLV and TLU have recently gained interest for the evaluation of [^177^Lu]Lu-PSMA-617 therapy and have also been found to correlate to overall survival [33–35]. Seifert et al. [35] found that TLV for the total tumour burden, pelvic lymph node metastases, distant lymph node metastases, bone metastases and visceral metastases were all significantly correlated to overall survival in a cohort of patients with varying indications for performing the PSMA PET–CT, including primary staging, biochemical recurrence, castration-sensitive metastatic disease and metastasised castration-resistant prostate cancer. Thus, it may be increasingly important in the future to obtain fully automatic calculations of tumour burden at different locations. Our method obtained measurements of TLV and TLU for all tumour locations that were strongly or very strongly correlated with manual measurements and in similar ranges as the correlations between two manual measurements.

Some limitations exist. This study was only trained on [^18^F]PSMA-1007 PET–CT scans, and performance of the AI model if other radiopharmaceuticals are used is not known. The PET–CT scans all came from a single hospital, which limits the generalisability of the model. Most of the manual segmentations used for training of the AI model were performed by one nuclear medicine physician. In addition, we have not trained the AI model to detect very rare locations of metastases, for example, lung or liver metastases (too few examples in our study group). Most of the patients in our training group had a low-disease burden. Our AI model might perform worse in patients with a very high tumour burden. Finally, no prospective evaluation of the AI model exists. We are currently working on an even larger AI-based model, including PET–CT scans with other PSMA radiopharmaceuticals scanned at other hospitals.

## Conclusions

By doubling the training dataset and refining the CNN architecture, we achieved a substantial improvement in the performance of our fully automated AI-based method for detecting and quantifying prostate tumour and suspected lymph node and bone metastases on [^18^F]PSMA-1007 PET–CT images. In particular, the PPV increased markedly, presenting a critical advancement toward clinical applicability. To promote transparency and facilitate further research, we have made our AI model freely available to the scientific community at www.recomia.org. We encourage independent validation across diverse clinical settings and imaging protocols to assess generalizability and support future integration into clinical workflows.

## Supplementary Information

Additional file1

## Data Availability

The datasets generated during the current study are available from the corresponding author on reasonable request. The AI-based model developed in this study is freely available for research at www.recomia.org.

## Abbreviations

AI: Artificial intelligence
CNN: Convolutional neural network
CT: Computed tomography
PET: Positron emission tomography
PPV: Positive predictive value
PSA: Prostate specific antigen
PSMA: Prostate specific membrane antigen
TLV: Total lesion volume
TLU: Total lesion uptake
