# Supplementary material for: A fully automated AI-based method for tumour detection and quantification on [18F]PSMA-1007 PET–CT images in prostate cancer
Source: EJNMMI Phys. 2025 Aug 20;12:78. doi: 10.1186/s40658-025-00786-9 (PMC12367631; doi:10.1186/s40658-025-00786-9)

**Supplementary Figures**

**Supplementary Figure 1.** Scatter plots of the correlation between TLV in prostate tumour/recurrence, suspected lymph node metastases and bone metastases measured by the AI and human readings.


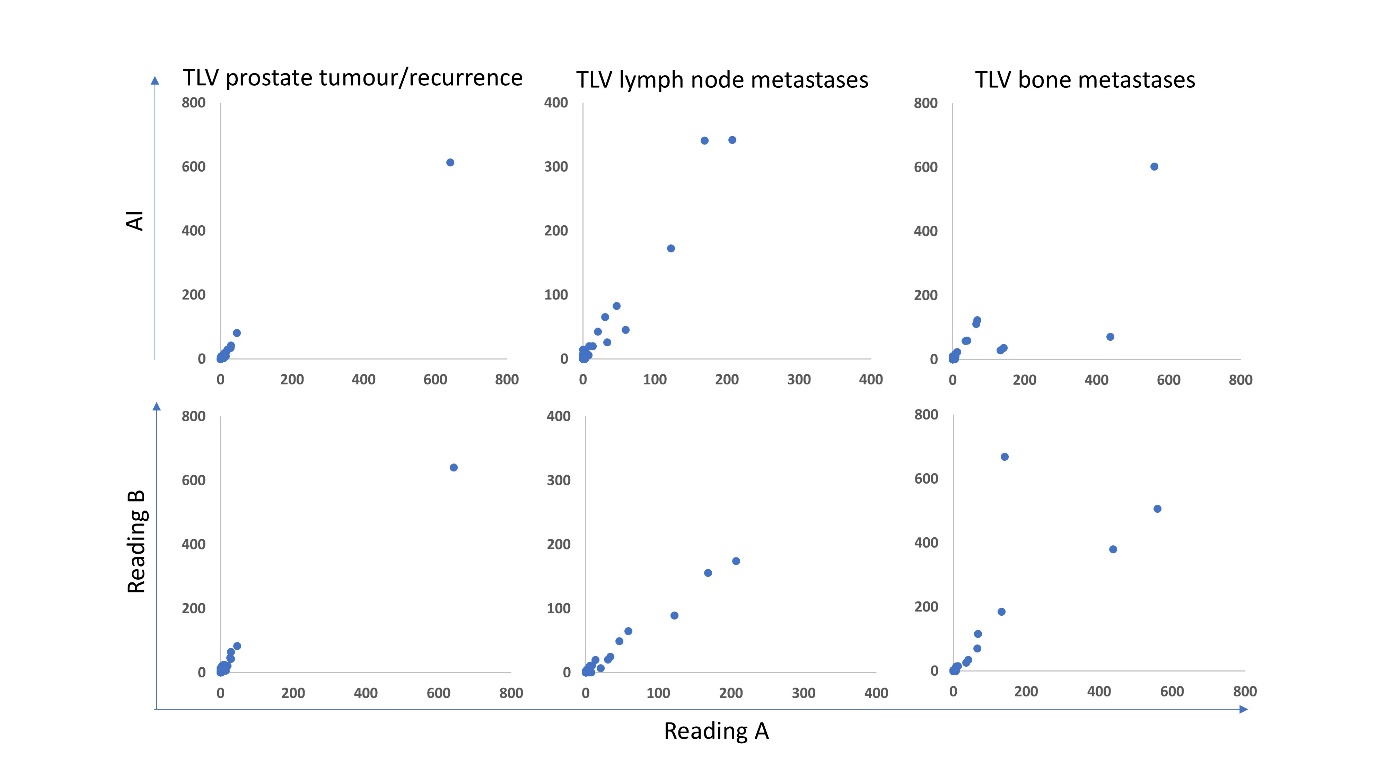


**Supplementary Figure 2.** Scatter plots of the correlation between TLU in prostate tumour/recurrence, suspected lymph node metastases and bone metastases measured by the AI and human readings.


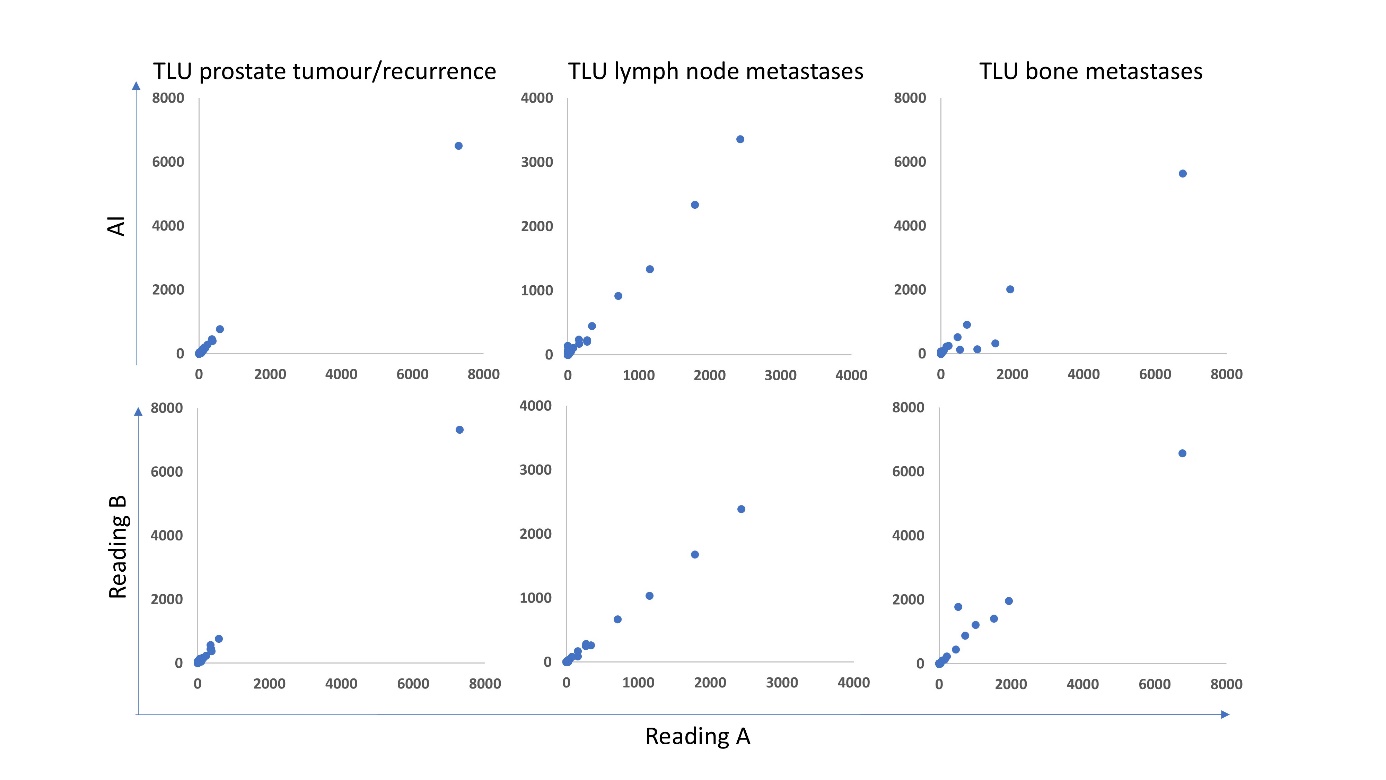

Supplement: Supplementary file 1 — Additional file1 [file 40658_2025_786_MOESM1_ESM.docx]
